# Notes on Medical Matters in Paris

**Published:** 1846-12

**Authors:** David W. Yandell

**Affiliations:** Louisville, Ky.


Art. IV.—*Notes on Medical Matters in Paris.* By David W. Yandell, M.D., of Louisville, Ky.

Paris, October 1st, 1846.

The regular course of lectures in the *Ecole de Médecine* are just about to open, and while as yet I am not in possession of a sufficient amount of matter strictly medical to fill a letter, I propose to occupy a little space in the Journal with a description of the *Garden of Plants.* This wonderful assemblage of objects, relating not only to our profession but to nearly every branch of natural history, has great interest for the medical man. In the close of my letter I shall be able to present a few items of medical news.

*Jardin des Plantes.*—Louis XIII was persuaded by his physicians, Herouard, and Guy de la Brosse, to establish the Jardin des Plantes. In 1739 the celebrated naturalist Buffon was appointed superintendant, and through his unwavering devotion to its interests many valuable additions were made to the garden, and many illustrious names, among whom were A. Petit, Faujas de St. Fond, Winslow, Fourcroy, and Daubenton, were placed upon the list of its patrons and contributors. During the Revolution, when the universities, the faculties of medicine, law, etc., were suppressed, it was feared that the garden would suffer from the proscription, but as it was looked upon as national property, open to all classes of visitors, and intended for the culture of medicinal plants, and the laboratory to be a manufacture of saltpetre, it was untouched. Throughout the Reign of Terror the garden was greatly neglected and crippled for the want of funds.

In 1814–'15 it was again endangered by the foreign troops; it was protected, however, by a special convention. Since that time the institution has been supported by the liberality of the State; large sums are annually voted for both the professors and pupils, the former of which are almost constantly occupied in and about the gardens, cabinets, lecture-rooms, and museums, and about eighteen hundred students annually listen to the courses of lectures delivered within the amphitheatre, laboratory, &c.

The garden is under the control of the Minister of the Interior, and consists of—1st. A botanical garden, with spacious hot-houses and green-houses. 2d. Several galleries containing collections, scientifically arranged, of the different kingdoms of nature. 3d. A gallery of comparative anatomy. 4th. A menagerie of living animals. 5th. A library of natural history. 6th. An amphitheatre, laboratories, &c. The lectures are all public and gratuitous; they begin in April and continue to the latter part of autumn; they are delivered early in the morning, and embrace various subjects connected with science. The amphitheatre is capable of holding twelve hundred persons. The number of species of plants cultivated in the botanical department is upwards of 12,000. Baron Cuvier arranged the cabinet of comparative anatomy, which I suppose surpasses any other in the world. Upon the first floor are the skeletons of the whale tribe; various marine animals; skeletons of every variety of the human species; skulls ranging from those of the lower animals up to man; large numbers of rooms up stairs are devoted to the dissections of fishes, reptiles, and birds, and some specimens of the human body. There are detached bones for the use of the students; series of the large bones and vertebræ of different animals; skeletons of quadrupeds; skeletons of birds, reptiles and fishes; series of teeth, commencing with those of the horse and ending with those of fishes; larynx and trachea, and hyoid bones of birds and quadrupeds. In phials are a series of brains and eyes; the bones of the ear of animals, from man to reptiles; a series of hearts of mammalia, reptiles and fishes; a wax model of the viscera of the hen, exhibiting the several periods of the formation of the egg; fœtal preparations of oviparous and viviparous animals; preparations of different orders of mollusca, zoophytes, articulated animals and shell-fish in wax. Besides these there are preparations of the muscles and viscera; skulls and casts of distinguished individuals; heads, in wax, by M. Calenzuoli, of Florence, representing the lymphatic and nervous system; fossil remains, &c.

In the Gallery of Zoology there is a collection of upwards of 2000 reptiles, embracing more than 500 species, divided into four orders; namely, chelonians, or tortoises; saurians, or lizards, comprehending crocodiles, etc.; ophidians, or serpents; and batrachians, as toads, frogs, &c. Also articulated animals without vertebræ, to the number of 25,000 species. The collection of birds is very complete, containing 2,500 species, and about 10,000 specimens. The number of species of fishes is the same, though there are only 5000 specimens; the number of mammalia is something like 500 species and 2000 specimens. Altogether the number of specimens of the animal kingdom is 200,000, and so beautifully and systematically arranged, that, beginning with the sponge, you may follow nature through her various gradations till she arrives at man.

The busts of Guy de la Brosse, Baron Cuvier, and others are in the building; a marble statue of Buffon also is in the gallery of zoology. The library, composed of works on natural history, has opposite its entrance a bust of Fourcroy; it contains 30,000 books and 15,000 pamphlets, but by far its most precious part are the manuscripts commenced in 1635, numbering now 90 port-folios, containing more than 6000 drawings of fruits and flowers upon vellum, and valued at two millions of francs.

There are in the mineralogical and geological gallery upwards of 60,000 specimens. The mineralogical collection is divided into the four grand classes,—1. earths containing an acid; 2, inflammable substances; 3, metals; 4, earthy substances or stones. Of the first and last there are many beautiful specimens, among them a vase of the brecciated porphyry of the Vosges, a large group of crystals of colorless quartz, &c., numerous cups of chalcedony, lapis lazuli, &c., &c.

Among the metallic substances are a great many aerolites. In the collection of inflammable substances are some pieces of yellow amber which contain insects imbedded while it was in its liquid state; a series of diamonds, &c.

The geological specimens are large, and the collection of fossils very complete. Valliant founded the botanical gallery. The herbal contains upwards of 50,000 species. The two cabinets of the fungi in wax, which was presented to the museum by Charles X and the Emperor of Austria, is very perfect, and its value is set down at 20,000 francs. M. Brongniart has arranged a collection of fossil plants from the coal formations, which is scientifically done and very interesting.

Of the menagerie it is hardly worth while to speak, save probably to say that it affords the student of natural history every opportunity to study the habits of a very large number of animals.

There are no animals in the collection that attract such crowds as the monkies do. They are provided with a commodious circular building of wire, and go to the garden when you will there are always around this establishment a number of Frenchmen, women, and children, enjoying as much as the monkies do themselves their gambols, quarrels, courtships, and races. In a collection of animals so large as this it is not to be expected that all the specimens will be perfect or healthy, and many of the poor creatures look miserably enough. The snake tribe, whose apartments are warm and protected from vicissitudes of temperature, are in good condition. This is a mere skeleton of a description of the garden, about which a volume might be written; but for the present it must suffice.

M. Marchal de Calvi, of the hospital of Val-de-Grace, has been employing for some time past iodine in a new form. The iodine is dissolved in oil, in the proportion of 1 grain to 18 grains. He takes afterwards a certain fixed quantity of the solution, mixes it with gum in a mortar, and forms an emulsion.

M. Marchal commences by prescribing one grain of iodine, or eighteen grains of the oily solution. The dose may be gradually augmented to six grains. Notwithstanding so large a dose has been used no unpleasant effects have been produced on the digestive tube. The patients preserve their appetite, and digestion is performed properly. This new preparation has been used with remarkable success in many cases of scrofulous swellings of the glands that had attained very great volume. Iodine in this form remains for a much longer time in the economy than the iodine with potassa. In place of iodide of potassium, M. Marchal uses the iodide of sodium, because he thinks it more active, from its containing a greater equivalent of iodine than the preparation containing potassa.

Dr. Desmarres reccommends in strong terms an anti-ophthalmic ointment prepared according to the following formula:

℞ Red precipitate 2 ä 4 grains.
Pulverised camphor 3 grains.
Olive oil 1 drop.

Mix and pulverise with care for a considerable length of time, afterwards add of fresh butter which has been washed in hot water, 54 grains. Make a perfectly homogeneous ointment.

It is used with great advantage in chronic inflammation of the transparent cornea, improperly called ophthalmia, among scrofulous individuals. Dr. D. has also prescribed it with much success in cases of spots not yet organised upon the cornea. The mode of employing it consists in applying in the evening just before going to bed a piece of the ointment the size of a grain of wheat upon the free borders of the eye lids.

Before the Academy of Sciences at its meeting on the 21st of September, Dr. Legrand spoke of the *action of gold upon the digestive organs.* The preparations of gold he believes are often of signal benefit to correct the gradual enfeeblement of the digestive organs, in old persons, in cases where quinine had effected no good. He mentioned the case of a woman 87 years of age, who was suffering from extreme debility, loss of appetite, &c. Syrup of quinine had been tried without advantage. Dr. Legrand prescribed 5 grains of gold mixed with ℥iv. of apple jelly. In sixteen days the dose of gold was augmented from the third of a grain per day to five grains. In twenty-five days the patient had taken 54 grains of gold, and the good was very perceptible; she had recovered much of her strength, her appetite was restored, and her food was taken with pleasure. A suspension of the remedy led to a return of the indisposition. The medicine was again given in even larger quantities than before, and resulted in the permanent cure of the patient. More recently the author has used another preparation of gold in a man 63 years old, who had been debilitated by antiphlogistic treatment, with similar success. In this case the preparation in question was the perchloride of gold and soda, with the powder of iris, which was rubbed upon the tongue.

The Duke of Dondeauville, in Germany, announces that he has discovered a remedy for the bite of rabid dogs. It consists of several glasses, one only at a time, of an infusion made of a small quantity of euphorbia villosa, veratrum album, polygonum hydropiper, and helleborus vulgaris. The plants must be gathered in May, early in June, or in September. While the infusion is preparing, the wound is to be washed with vinegar and water. Besides the virtue of healing, this remedy possesses another which is even more remarkable; it detects whether the dog that inflicted the bite was really rabid. If he was mad, the third, or at most, the fourth dose produces violent vomiting; if not mad the remedy possesses no such power.

